# Photobiomodulation Therapy Improves Repair of Bone Defects Filled by Inorganic Bone Matrix and Fibrin Heterologous Biopolymer

**DOI:** 10.3390/bioengineering11010078

**Published:** 2024-01-13

**Authors:** Maria Fernanda Rossi Vigliar, Lais Furlaneto Marega, Marco Antonio Hungaro Duarte, Murilo Priori Alcalde, Marcelie Priscila de Oliveira Rosso, Rui Seabra Ferreira Junior, Benedito Barraviera, Carlos Henrique Bertoni Reis, Daniela Vieira Buchaim, Rogerio Leone Buchaim

**Affiliations:** 1 Graduate Program in Anatomy of Domestic and Wild Animals, Faculty of Veterinary Medicine and Animal Science, University of Sao Paulo (FMVZ/USP), Sao Paulo 05508-270, Brazil; mariafernandarossiv@alumni.usp.br (M.F.R.V.); danibuchaim@alumni.usp.br (D.V.B.); 2 Department of Biological Sciences, Bauru School of Dentistry, University of Sao Paulo (FOB/USP), Bauru 17012-901, Brazil; dra.laismarega@gmail.com (L.F.M.); marcelierosso@alumni.usp.br (M.P.d.O.R.); dr.carloshenriquereis@usp.br (C.H.B.R.); 3 Department of Dentistry, Endodontics and Dental Materials, Bauru School of Dentistry, University of Sao Paulo (FOB/USP), Bauru 17012-901, Brazil; mhungaro@fob.usp.br (M.A.H.D.); murilo.alcalde@usp.br (M.P.A.); 4 Center for the Study of Venoms and Venomous Animals (CEVAP), Sao Paulo State University (University Estadual Paulista, UNESP), Botucatu 18610-307, Brazil; rui.seabra@unesp.br (R.S.F.J.); benedito.barraviera@unesp.br (B.B.); 5 Graduate Programs in Tropical Diseases and Clinical Research, Botucatu Medical School (FMB), Sao Paulo State University (UNESP–University Estadual Paulista), Botucatu 18618-687, Brazil; 6 Postgraduate Program in Structural and Functional Interactions in Rehabilitation, Postgraduate Department, University of Marilia (UNIMAR), Marilia 17525-902, Brazil; 7 Medical School, University Center of Adamantina (UNIFAI), Adamantina 17800-000, Brazil

**Keywords:** bone regeneration, bone repair, biomaterial, fibrin tissue adhesive, low-level laser therapy, photobiomodulation therapy, scaffolds, fibrin sealant, fibrin

## Abstract

Biomaterials are used extensively in graft procedures to correct bone defects, interacting with the body without causing adverse reactions. The aim of this pre-clinical study was to analyze the effects of photobiomodulation therapy (PBM) with the use of a low-level laser in the repair process of bone defects filled with inorganic matrix (IM) associated with heterologous fibrin biopolymer (FB). A circular osteotomy of 4 mm in the left tibia was performed in 30 Wistar male adult rats who were randomly divided into three groups: G1 = IM + PBM, G2 = IM + FB and G3 = IM + FB + PBM. PBM was applied at the time of the experimental surgery and three times a week, on alternate days, until euthanasia, with 830 nm wavelength, in two points of the operated site. Five animals from each group were euthanized 14 and 42 days after surgery. In the histomorphometric analysis, the percentage of neoformed bone tissue in G3 (28.4% ± 2.3%) was higher in relation to G1 (24.1% ± 2.91%) and G2 (22.2% ± 3.11%) at 14 days and at 42 days, the percentage in G3 (35.1% ± 2.55%) was also higher in relation to G1 (30.1% ± 2.9%) and G2 (31.8% ± 3.12%). In the analysis of the birefringence of collagen fibers, G3 showed a predominance of birefringence between greenish-yellow in the neoformed bone tissue after 42 days, differing from the other groups with a greater presence of red-orange fibers. Immunohistochemically, in all experimental groups, it was possible to observe immunostaining for osteocalcin (OCN) near the bone surface of the margins of the surgical defect and tartrate-resistant acid phosphatase (TRAP) bordering the newly formed bone tissue. Therefore, laser photobiomodulation therapy contributed to improving the bone repair process in tibial defects filled with bovine biomaterial associated with fibrin biopolymer derived from snake venom.

## 1. Introduction

Bone tissue contains cellular elements such as osteoblasts, osteocytes and osteoclasts, as well as organic and inorganic extracellular matrix. When injured, as in cases of fractures, it has repair capacity with local vascular proliferation. This event is stimulated by the production of chemical mediators by macrophages and platelets and, through these vessels, the migration of undifferentiated or poorly differentiated cells occurs, which subsequently differentiate and take on their function in the tissue, such as the production of extracellular matrix. The newly formed vessels and undifferentiated cells are supported by a three-dimensional fibrin network formed by the blood clot or exudate [1,2].

Bone repair of tibial fractures, because it is endochondral ossification, occurs in a complex manner and can be divided into three main phases: inflammation, repair and remodeling [3,4,5], generally occurring outside the periosteum, in regions immediately adjacent to the fracture site and mechanically less stable [6,7,8]. For repair to occur effectively, it depends on a number of factors, such as care taken with the surgical environment, surgical margins, vascular proliferation, migration of undifferentiated cells, the presence of a fibrin network and, when possible, preserving the periosteum and endosteum [9,10,11]. When bone repair does not take place in the injured area, the lesion will be filled with fibrous connective tissue and the tissue will not recover its original functionality [1,12,13]. In cases where one of these factors cannot be preserved, biomaterials can be used to recompose the bone tissue [14,15,16,17].

Biomaterial grafts are important in the fields of medicine and dentistry, and are mainly indicated for the reconstruction of bone tissue in areas of accentuated loss, due to the consequent impairment of function, morphology and registration of biological tissues [18,19,20,21]. There is a wide variety of graft materials, such as autogenous (from the recipient itself), homogeneous (from another non-recipient individual, of the same species), heterogeneous (from other species) and allogeneic (from tissues, but not living) [22,23,24]. They can have osteoinductive characteristics, with the ability to induce the migration of undifferentiated cells to the repair site, osteoconductive with the ability to provide a structural framework for neoformed vessels and undifferentiated cells, osteogenic with the ability to generate growth through the transfer of viable cells, with autografts being the only ones with this ability, or osteopromotive, with the ability to carry out local isolation, selecting cell proliferation, mainly osteoblasts [25,26,27].

Autograft is considered the gold standard. It is the only osteogenic material with great osteoinductive and osteoconductive capacity, as well as advantages such as less chance of infection, no rejection, lower financial costs, greater predictability of success and a low rate of resorption; however, they require a donor site, increasing patient morbidity, longer surgical time and, in some cases, the volume required at the recipient site is limited [28,29,30]. Xenografts have been used in the treatment of osteotomies, arthrodesis and fractures, in the restoration of bone loss by promoting the induction of bone formation and providing a mechanism for vascular support (osteoconduction) and bone growth [31,32,33]. The advantages of using heterogeneous biomaterials are based on the need for a single surgical site and, therefore, causing less postoperative morbidity and shorter surgical time, and also avoid weakening the donor region [34,35,36]; a disadvantage is the possibility of disease transmission. Osteosubstitute materials result in biocompatible and partially absorbable biomaterials, favoring the repair of the bone defect [37,38,39].

Bovine bone processing can result in two different types of materials: inorganic and organic. The inorganic is free of proteins and cells and is characterized by a high content of hydroxyapatite. Bonefill^®^ inorganic bone matrix (Bionnovation^®^, Bauru, Brazil) is extracted from bovine femur completely denatured, that is, it does not have an organic portion that can induce immunogenic processes in the body [40]. This material is easy to manipulate for installation in the surgical bed. It is suggested that it is capable of providing new bone formation and growth, resulting in neoformed tissue with a firm clinical consistency and high cellular activity [41,42].

More specifically in the clinical applicability of biomaterials, in a randomized controlled clinical study, two biomaterials were compared, a synthetic bone substitute (Straumann Bone Ceramics^®^, Institut Straumann AG, Basel, Switzerland) and a bovine-derived xenograft (Bio-Oss^®^ deproteinized bovine bone mineral, Geistlich Pharma AG, Wolhusen, Switzerland), evaluating their potential to preserve the dimensions of the alveolar ridge after tooth extraction. Both biomaterials were effective in partially preserving the width and interproximal bone height of the alveolar ridge [43].

Fibrin sealants can be associated with biomaterials acting as a support system in an attempt to improve the repair of bone lesions [44,45]. A new fibrin sealant purified from snake venom (currently called Heterologous fibrin biopolymer, FB) has been produced by Brazilian researchers with the advantage of not containing human blood. It has the characteristics of a biodegradable sealant, with good adhesion properties and without transmitting infectious diseases [46,47,48].

In addition, attempts are being made to optimize bone regeneration through physical methods, promoting more effective morphophysiological rehabilitation in a shorter period. One of the recent techniques with reports of success in this area is low-level laser therapy—LLLT [49,50,51]. LLLT in bone tissue has been shown to be effective mainly in modulating the inflammatory response and accelerating the tissue repair process [52,53,54]. Given the lack of studies, associating the biomaterials mentioned above (mineralized inorganic bone matrix and FB) with PBM, the aim of this pre-clinical study was to analyze the effects of photobiomodulation therapy on the repair process of surgically created bone defects in the tibia of rats, filled with mineralized inorganic bone matrix associated with heterologous fibrin biopolymer.

## 2. Materials and Methods

### 2.1. Experimental Design

Thirty animals were kept and operated on in the animal house of the Bauru School of Dentistry (University of São Paulo, Bauru, Brazil), according to the inclusion criteria: adult male rats (*Rattus norvegicus*), Wistar strain, weighing approximately 310 g and 90 days old. This experimental protocol was approved by the Ethics Committee for the Use of Animals of the Bauru School of Dentistry, University of São Paulo (FOB/USP), under protocol CEEPA 009/2016.

This study followed the ARRIVE (Animal Research: Report of in vivo Experiments) checklist, assessing the rigor of the methodology and replication of the methods and results [55,56]. Throughout the experiment, all the animals were monitored for any signs that they were apathetic, in pain or depressed, possibly aggressive or hyper-excitable, always compared to their usual behavior. Changes in gait and locomotion, posture, facial expression, water and food consumption were also assessed, and clinical symptoms monitored. In addition, this study was ethically based on the number of animals (*n*) and their random distribution in the experimental groups, the principle of the 3R’s, in the principles of “replacement, reduction and refinement” in the use of animals.

The preference for male rats was due to an attempt to avoid the influence that sex hormones have on bone tissue, which can have an inhibitory action on the periosteum in bone formation, a fact attributed to estrogen in females [57,58]. In the bioterium, the animals were separated into cages with 4 animals in each, with artificial lighting monitored by a timer, which controlled the light/dark cycle of 12 h each, as well as air conditioning, set at 23 °C, and an exhaust fan. After the experimental surgery, the animals were individualized in their cages.

**Figure 1:**
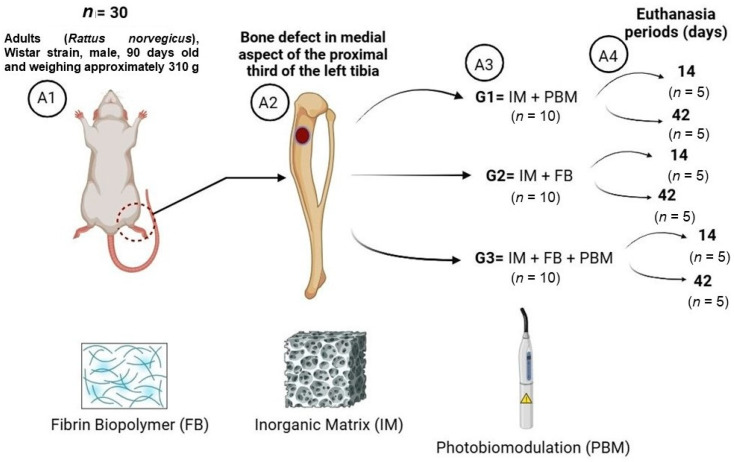
Experimental design. (**A1**) Total number of animals and inclusion criteria. (**A2**) Monocortical osteotomy in the medial aspect of the proximal third of the tibia. (**A3**) G1 = IM + PBM: Group 1, defects that received with Inorganic Matrix (IM) and Photobiomodulation (PBM). G2 = IM + FB: Group 2, defects that received with Inorganic Matrix (IM) and fibrin biopolymer (FB). G3 = IM + FB + PBM: Group 3, defects that received Inorganic Matrix (IM), fibrin biopolymer (FB) and Photobiomodulation (PBM). (**A4**) The animals were euthanized at 14 and 42 days, with five animals in each group and at each period.

The animals were divided into three groups (Figure 1):

(1) G1 (*n* = 10): Inorganic Matrix + Photobiomodulation (IM + PBM);
(2) G2 (*n* = 10): Inorganic Matrix + fibrin biopolymer (IM + FB);
(3) G3 (*n* = 10): Inorganic Matrix + fibrin biopolymer + Photobiomodulation (IM + FB + PBM).

The number of animals per group (*n* = 10) was based on previous studies with the necessary quantity to favor statistical data, maintaining the policy of reducing the use of animals in research. Euthanasia periods of 14 and 42 days were also used in previous studies, with 14 days being used to evaluate PBM in the initial stages of inflammation and 42 days for the possible increase in the percentage of bone formed [23,59,60].

### 2.2. Experimental Surgery

For the experimental surgery, the rats underwent general anesthesia with intramuscular injection of tiletamine hydrochloride and zolazepam hydrochloride (10 mg/kg, Telazol^®^, Fort Dodge Laboratories, Overland Park, KS, USA), trichotomy of the site and disinfection with 10% topical iodine solution. Next, a 20 mm long linear incision was made in a craniocaudal direction on the left pelvic limb using a No. 15 scalpel blade, sectioning the skin and muscle fascia to expose and disclose the muscle tissue surrounding the tibia.

The incision reached the periosteum, allowing it to be correctly separated in an anteroposterior direction, providing the necessary visualization of the operative field. A spherical drill made of No. 8 steel, mounted in contra-angle (Driller^®^, São Paulo, Brazil) coupled to an electric micromotor (Driller^®^ BLM 600 Baby, São Paulo, Brazil), at low speed (1500 rpm) and torque of 45 N·cm, was used to prepare a cavity approximately 4 mm in diameter and deep enough to reach the bone marrow. This procedure was carried out with abundant irrigation of 0.9% saline solution.

In all the animals, the surgical cavity was filled with Bonefill^®^ inorganic bone matrix. Bonefill^®^ is sold commercially (Bionnovation^®^, Bauru, Brazil), produced by decalcification of the cortical portion of bovine femur, and registered by the Brazilian Health Regulatory Agency (ANVISA10392710012), with a Ca/P ratio of 1.67, porosity ~65–80%, average pore size ~100–600 μm and granule size 0.10–0.6 mm.

In the G2 animals, Bonefill^®^ was mixed with fibrin biopolymer. The constituents of the heterologous fibrin biopolymer, its formula and forms of application are in accordance with patent number BR102014011432-7, issued on 6 July 2022, by the National Institute of Industrial Property of Brazil (INPI). At the time of use, the components of the FB were previously thawed, reconstituted, mixed and applied with micropipettes according to the following protocol: First was the thrombin-like enzyme fraction, fraction 1, derived from snake venom *Crotalus durissus terrificus* (15 µL), the second contains calcium chloride diluent (15 µL) and the last (fraction 2) fibrinogen extracted from buffalo blood (30 µL) *Bubalus bubalis*. The FB production process is described in detail in Barros et al. (2009) [61].

In the three groups of this study, in each of the animals, the defect created surgically in the region of the proximal metaphysis of the tibia was filled with 0.012 g of Bonefill^®^ biomaterial, previously weighed on an analytical balance (Micronal^®^, Precision Equipment, São Paulo, Brazil). In Group 1 (IM + PBM), the biomaterial was incorporated into saline solution before insertion into the defect. In Groups 2 (IM + FB) and 3 (IM + FB + PBM), a biocomplex was formed, as FB was associated with Bonefill^®^.

For the animals in the G1 and G3, a continuous pulse GaAlAs (gallium–aluminum–arsenide) laser photobiomodulation was applied, with a wavelength of 830 nm, 30 mW of optical output power, energy density of 6.2 J/cm^2^, area of contact of 0.116 cm^2^, for 24 s/applied site, in two points of the operated site. The pen was kept in contact with the animal’s skin, making a total application time of 48 s, performed immediately after surgery and three times a week, until the period corresponding to euthanasia. The PBM parameters used in this study were based on previous studies by our group of researchers, with favorable results in increasing new bone formation in the tibia [23] or calvaria of rats [62] (Table 1).

The removed tissues were repositioned and the sutures with 4–0 silk thread (Ethicon^®^, Johnson and Johnson Company, São Paulo, Brazil), in separate single stitches, were completed. After the surgery, the animals were given the analgesic paracetamol at a dose of 200 mg/Kg dissolved in the drinking water for 3 days.

### 2.3. Euthanasia and Tissue Collection

After 14 and 42 days post-surgery, five animals from each group were euthanized in a quiet environment away from the other animals. The barbiturate (thiopental) was used at a dose of (150 mg/kg) as follows: Thiopental Sodium 2.5%, intraperitoneally, IP, applied to the lower left quadrant of the animal’s abdomen (associated with the local anesthetic lidocaine hydrochloride at a dose of 10 mg/kg). Once the animal was confirmed dead, it was placed in a white biohazard bag and sent for disposal.

The tibia was dissected, removed and fixed in 10% buffered formalin for 24 h, washed in running water for 12 h. The specimens were then sent for computerized microtomography and histological processing, with decalcification in a 10% ethylenediaminetetraacetic acid (EDTA) solution, changed every 7 days for the demineralization process, which was completed after 6 weeks.

To perform the surgical technique and apply PBM, with the aim of standardizing and standardizing all procedures between the experimental groups, each step was always performed by the same operator.

### 2.4. Computerized Microtomography

The specimens were positioned within a cylindrical acrylic tube and inserted into the SkyScan 1174v2 microtomograph (µ-CT Bruker microCT^®^, Kontich, Belgium), yielding images with a voxel size of 13.76 µm, and an angular increment of 0.73 degrees for each scan. Subsequently, the software NRecon^®^ 1.6.9 and DataViewer^®^ 1.4.4.0 were employed to execute the two-dimensional reconstruction and alignment analyses, respectively. To reconstruct the three-dimensional images, the software CTVox^®^ 2.4.0 r870 (Bruker microCT) was utilized.

### 2.5. Histological Processing

After microtomography and decalcification, the collected pieces were embedded in paraffin. With the blocks obtained, 5 µm longitudinal sections were made. The slides were stained with hematoxylin-eosin (HE), Masson’s trichrome, Picrosirius-red and immunohistochemistry for tartrate-resistant acid phosphatase (TRAP) and osteocalcin (OCN).

The semi-serial sections of the surgical site for each defect underwent evaluation using an Olympus^®^ BX50 light microscope (Olympus Corporation^®^, Tokyo, Japan). Photographic documentation was performed using 4× and 10× magnification objectives, employing both HE staining and Masson’s trichrome staining. A digital camera (Olympus^®^ DP 71, Tokyo, Japan) was utilized for capturing the images. This process was facilitated by image capture software DP Controller 3.2.1.276 (Olympus Corporation^®^, Tokyo, Japan), which was set with image dimensions of 4080 × 3072 pixels and a spot size of 0.1%.

Sections stained with Picrosirius-red were evaluated under conventional and 10× polarized light to determine the newly formed organic matrix quality over the experimental periods of defect repair. Images of the defect were obtained using a Leica DFC 310FX higher resolution digital camera (Leica, Microsystems^®^, Wetzlar, Germany) connected to the Leica DM IRBE confocal laser microscope and the LAS 4.0.0 capture system (Leica^®^, Microsystems, Heerbrugg, Switzerland). For the binarized image, the ImageJ^®^ software was used (version 1.41o, Java 1.6.0_10, Wayen Rasband, US National Institutes of Health, Bethesda, MD, USA).

For the qualitative histological description, the defect was considered in its entirety, analyzing the presence of inflammatory infiltrate, granulation tissue and type of bone formed (mature/immature).

### 2.6. Immunohistochemical Analysis

For immunohistochemistry analysis, two silanized slides with three sections from each animal were removed from the paraffin in xylene and hydrated in a decreasing series of ethanol. Antigenic recovery was carried out by immersing the histological slides in citrate buffer (Spring Bioscience^®^, Pleasanton, CA, USA) in a pressurized chamber (Decloaking chamber^®^, Biocare Medical, Concord, CA, USA) at 95 °C for 20 min. At the end of each stage of the immunohistochemical reaction, the histological slides were washed in 0.1 M buffered saline solution (PBS), pH 7.4. Subsequently, the slides were immersed in 3% hydrogen peroxide for 1 h and 1% bovine serum albumin for 12 h to block endogenous peroxidase and block nonspecific sites, respectively.

Histological sections were separated into two batches and each incubated with the following primary antibodies: goat-generated mouse anti-OCN (SC-18319, Santa Cruz Biotechnology^®^, Santa Cruz, CA, USA), and goat anti-TRAP goat-generated mouse (SC-30833, Santa Cruz Biotechnology^®^, Santa Cruz, CA, USA). They were then incubated with biotinylated secondary antibody for 2 h and then treated with streptavidin conjugated to horseradish peroxidase—HRP for 1 h (Universal Dako Labeled HRP Streptavidin-Biotin Kit^®^, Dako Laboratories, Santa Clara, CA, USA). Development was performed using 3,3’-diaminobenzidine tetrahydrochloride as a chromogen (DAB chromogen Kit^®^, Dako Laboratories, Santa Clara, CA, USA). Counterstaining with Harris Hematoxylin for OCN and TRAP was performed. Then, the slides were subjected to dehydration in ethanol, clearing in xylol and covered with mounting medium (Permount, Fisher Scientific^®^, San Diego, CA, USA) and glass coverslips. As a negative control, the specimens were submitted to the procedures previously described, suppressing the use of the primary antibody.

Then, the histological slides were evaluated by optical microscopy (Olympus model BX50^®^, Tokyo, Japan) with amplitudes of ×10 (Olympus Corporation^®^, Tokyo, Japan) and photographs taken with the attached digital camera (Olympus DP 71^®^, Tokyo, Japan) for qualitative analysis.

### 2.7. Morphometric Evaluation

The quantification of bone neoformed was made from images captured with a 4× objective using the planimetry point counting method. Therefore, an 88-point grid was superimposed on the histological image captured from each animal in the photomicroscope, computing each point that was located on the newly formed tissue, and the total density was quantified by percentage occupancy (%) [23], scanning the entire image.

The size of the grid used was standardized at 13.2 × 9.6 cm, with a spacing of 1.2 cm between each point marked on a printed transparent sheet. The area density was calculated using the equation: D = ΣPN/PT × 100, where PN indicates the number of overlapping points in the new bone tissue and PT the total number of points included in the overlapping grid. The percentage value obtained was related to the average of all morphometrically analyzed animals.

Two slides from each region of the defect (proximal, middle and distal) from each animal/group were included for the morphometric analysis, with three sections on each histological slide. The measurements obtained were imported to Microsoft Excel^®^ (Microsoft, Redmond, WA, USA).

### 2.8. Statistical Analysis

The quantitative data was subjected to statistical analysis using the GraphPad Prism 8 program (GraphPad^®^ Software 2018, San Diego, CA, USA). An unpaired *t*-test was used to analyze the influence of time on neoformed bone (percentage, %) in each experimental group, and an ANOVA for independent samples and Tukey’s post hoc test were used to compare the neoformed bone in each experimental period. Values of *p* < 0.05 were considered statistically significant in all analyses (*n* = 5 for each period and group). Bartlett’s test was used to analyze normality.

## 3. Results

In relation to animal experimentation, there were no complications that required intervention beyond what was planned, with medication and monitoring. There was no loss of any animal, from any group. The superficial tissues of the operated area were repaired 14 days post-operatively.

### 3.1. Microtomographic Evaluation

The 2D and 3D radiographic reconstructions obtained through microcomputed tomography reveal that the biomaterial shares similarities with cortical bone, mainly in terms of radiopacity, challenging the process of quantitative analysis of newly formed bone. The greater the radiopacity, the greater the degree of mineralization.

**Figure 2:**
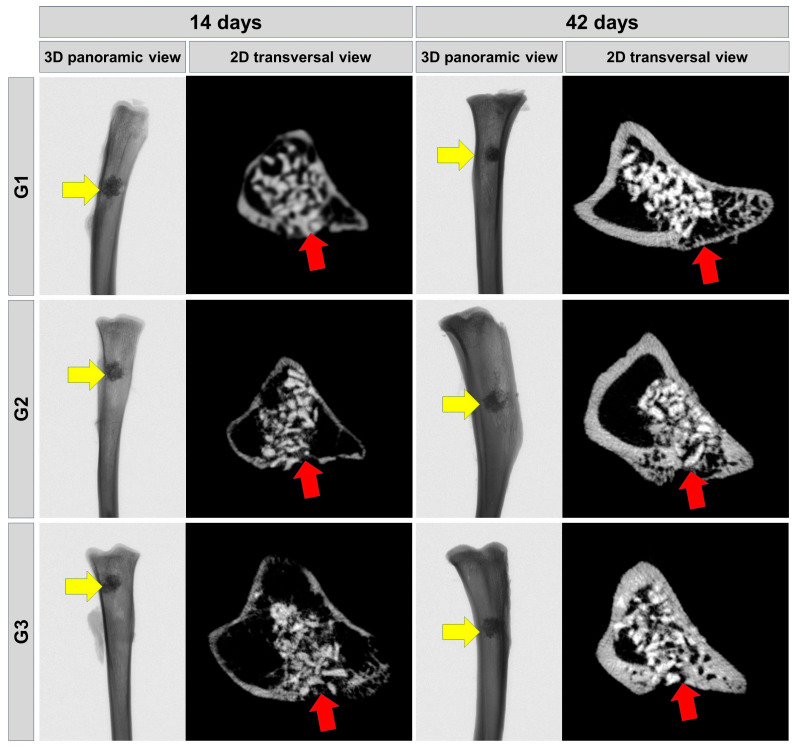
Longitudinal µ-CT images showing the repair process of the surgical defect with graft (biomaterial) and/or fibrin biopolymer with or without low-level laser photobiomodulation. Cortical with newly formed bone 3D panoramic view (yellow arrows) and 2D transverse view (red arrows). Two-dimensional transaxial sections at 14 days and 42 days, respectively. G1—Inorganic Matrix + Photobiomodulation (IM + PBM); G2—Inorganic Matrix + fibrin biopolymer (IM + FB); G3—Inorganic Matrix + fibrin biopolymer + Photobiomodulation (IM + FB + PBM).

In the 14-day trial period, it is clear that there is a monocortical bone defect with defined limits in all experimental groups, covered by the biomaterial. The volume of bone formed is greater at 42 days in all groups. In 42 days, the presence of newly formed bone covering the cortical area can be observed, with particles of biomaterials interspersed in this new bone formation, indicating a process of healing and bone maturation. In G1, the biomaterial particles are more evident, with little newly formed bone tissue and of lower radiopacity interspersing its particles, while in G2 and G3, the biomaterial is better integrated with the surrounding newly formed bone tissue, being more evident in G3 (Figure 2).

### 3.2. Histomorphological Analysis

In the period of 14 days, all groups showed a normal process of cortical bone remodeling, presence of biomaterial particles, loose connective tissue and isolated areas of new bone formation. In G3, there was a transition from loose connective tissue to dense connective tissue surrounding the xenograft and medullary tissue.

**Figure 3:**
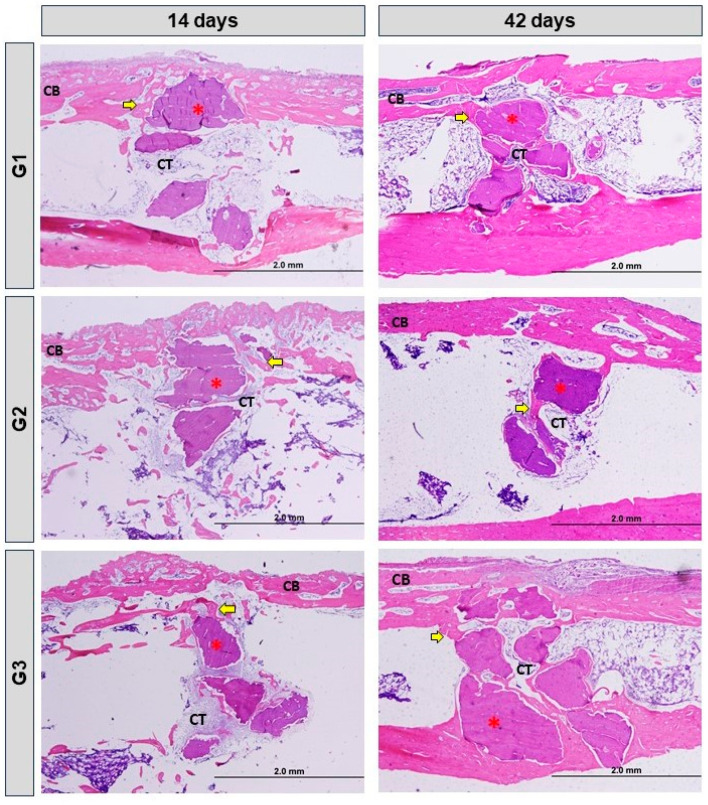
Longitudinal histological images of the bone defect in the tibia in the different groups: G1—Inorganic Matrix + Photobiomodulation (IM + PBM); G2—Inorganic Matrix + fibrin biopolymer (IM + FB); G3—Inorganic Matrix + fibrin biopolymer + Photobiomodulation (IM + FB + PBM), in the experimental periods, 14 and 42 days. The cortical defect is located in the upper part of all the images. Cortical bone (CB); Dense connective tissue (CT); Biomaterial particles (red asterisks); Immature bone (yellow arrows). Original magnification = 4×. 2.0 mm scales bar. HE stain.

**Figure 4:**
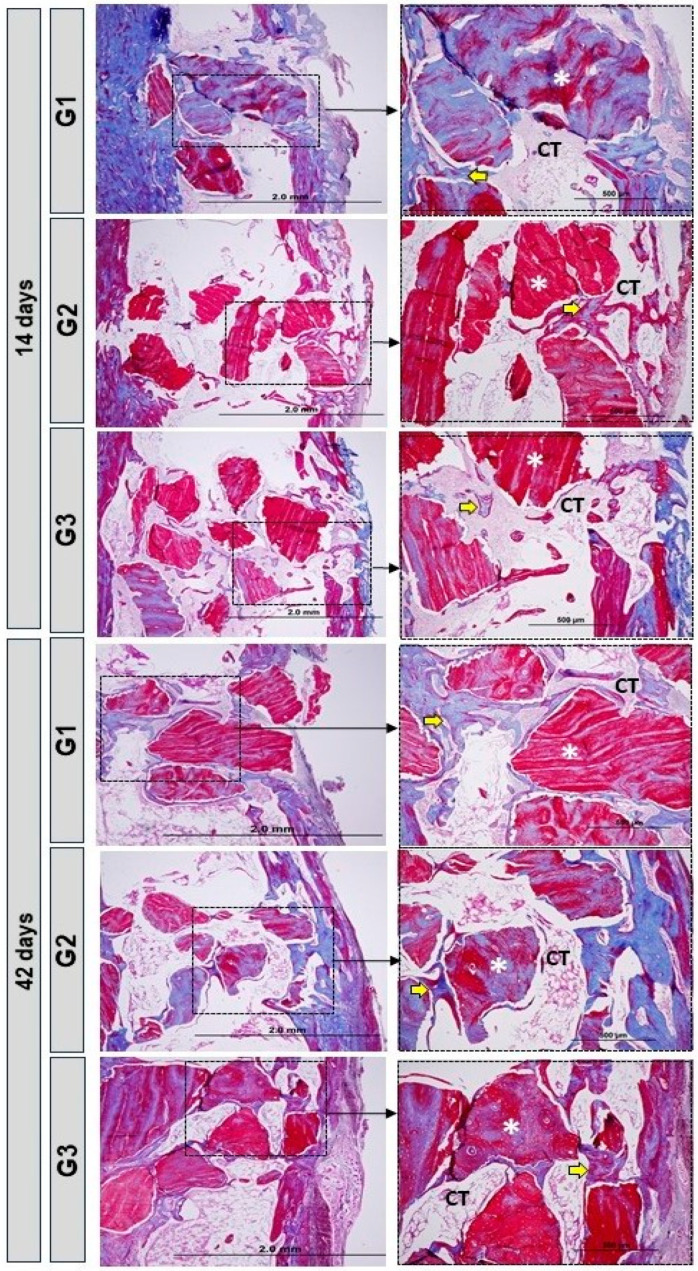
Longitudinal histological images of the bone defect in the tibia in the different groups: G1—Inorganic Matrix + Photobiomodulation (IM + PBM); G2—Inorganic Matrix + fibrin biopolymer (IM + FB); G3—Inorganic Matrix + fibrin biopolymer + Photobiomodulation (IM + FB + PBM), in the experimental periods, 14 and 42 days. The cortical defect is located in the upper part of all the images. Connective tissue (CT); Biomaterial particles (white asterisks); Immature bone (yellow arrows). Original magnification = 4× and 10×. 2.0 mm and 500 µm scales bar. Masson’s trichrome stain.

In 42 days, the groups showed irregularity on the surface of the biomaterial due to absorption, increase in bone formation around the particles. In G3, there was a greater area of bone formation with similarity in the physical aspect of bone maturation. All groups exhibited dense connective tissue and a more advanced stage of cortical bone remodeling, which is more evident in G3 (Figure 3 and Figure 4).

At 14 days, mainly due to the accommodation of the microenvironment to the biomaterial graft, inflammatory cells were observed but without reaction to the foreign body. At 42 days, mainly in G1 and G3, with the use of photobiomodulation, we did not show any local inflammatory reaction on the histological slides.

### 3.3. Birefringence Analysis of Collagen Fibers

In the images obtained from histology using Picrosirius-red staining, a red-orange appearance was evident in the newly formed bone tissue, characteristic of immature bone, with disorganized direction of the fine collagen fibers, especially at 14 days. In the groups with LLLT, there was a greater presence of yellowish-green fibers/mature bone, especially in G3. All groups presented biomaterial particles surrounded by a predominance of red-orange fibers, characteristic of the presence of immature bone.

**Figure 5:**
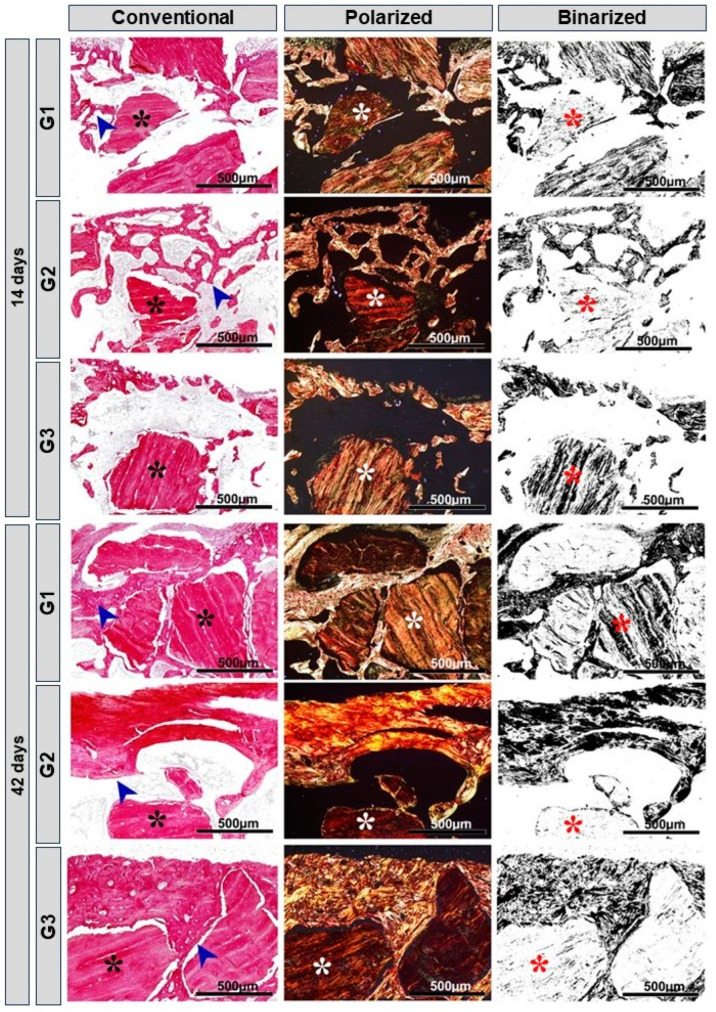
Histological images of the birefringence of collagen fibers in conventional, polarized and binarized microscopy exemplified by the G1 Inorganic Matrix + Photobiomodulation (IM + PBM), G2 Inorganic Matrix + fibrin biopolymer (IM + FB) and G3 Inorganic Matrix + fibrin biopolymer + Photobiomodulation (IM + FB + PBM) groups, at 14 and 42 days. Biomaterial (black, white and red asterisks) and new bone formation (blue arrow). Original magnification = 10×. Scale bars = 500 µm. Picrosiris-red stain.

At 42 days, all groups showed changes in birefringence in relation to 14 days, compatible with more organized bone tissue. In G3, conventional microscopy (blue arrow), organization and maturation were more evident (Figure 5).

### 3.4. Immunohistochemical Analysis

The immunohistochemical technique used in this study showed high specificity in detecting OCN (osteocalcin) and TRAP (tartrate-resistant acid phosphatase). The immunoreactive cells showed a dark brown color confined to different cell structures: TRAP—marking confined exclusively to the cell cytoplasm, and OCN—marking confined to the cell cytoplasm and, to a lesser extent, the extracellular matrix. Histological evaluation of bone mineralization and maturation responses was carried out using the osteocalcin antibody, which is marked in cells of the osteoblastic lineage, both in the mineralized and non-mineralized bone matrix, as well as the bone resorption response through TRAP, which marks osteoclasts that are active, together with the mineralized bone matrix.

**Figure 6:**
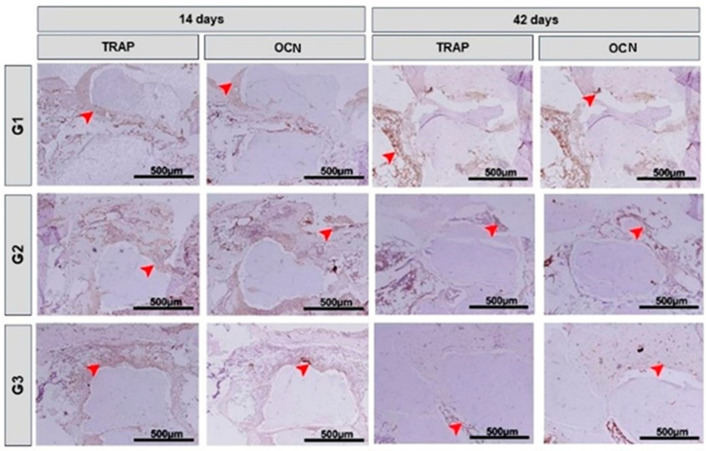
Histological images of the immunostaining pattern for TRAP and OCN inside the defect and in the tissues located close to the ruptured cortical in the G1 Inorganic Matrix + Photobiomodulation (IM + PBM), G2 Inorganic Matrix + fibrin biopolymer (IM + FB) and G3 Inorganic Matrix + fibrin biopolymer + Photobiomodulation (IM + FB + PBM) groups, at 14 and 42 days. Red arrows: immunostained cells. Counterstain: Harris Hematoxylin. Original magnification = 10×. Scale bars = 500 µm.

At 14 days, all experimental groups showed immunostaining for OCN close to the bone surface of the defect margins, in the connective tissue and on the surface of the newly formed bone tissue on the biomaterial particles. TRAP immunostaining was present in the newly formed bone around the biomaterial particles (Figure 6). At 42 days, groups G1, G2 and G3 showed immunostaining for OCN in the newly formed bone lamellae and in some fibroblasts. In the analysis of OCN and TRAP, a greater pattern of immunostaining could be observed in the G1 group when compared to the others. TRAP immunostaining patterns were higher in bone marrow tissue (Figure 6).

### 3.5. Morphometric Analysis

**Figure 7:**
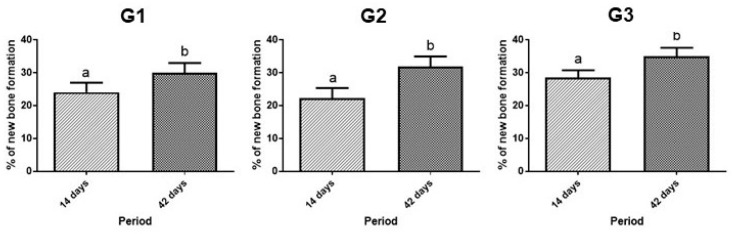
Percentage of bone neoformed (%) in groups G1, G2 and G3, in 14 and 42 days. a ≠ b, different lowercase letters, indicate difference with statistical significance. Unpaired *t*-test. Values mean ± standard deviation (*p* < 0.05).

In the analysis of the comparison of bone neoformed (%) in each group, between 14 and 42 days, there was a difference in the three groups. Within each group, when comparing 14 days with 42 days, there was a significant difference, and bone formation was always greater at 42 days (Figure 7 and Table 2).

**Figure 8:**
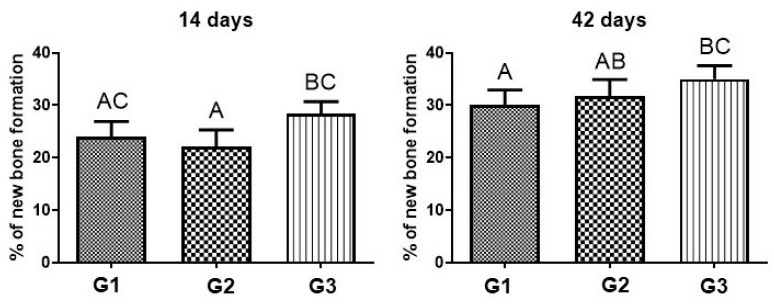
Percentage of bone neoformed (%) in groups G1, G2 and G3, in 14 and 42 days. A ≠ B ≠ C, different capital letters indicate difference with statistical significance. Values defined as the mean ± standard deviation (*p* < 0.05), ANOVA test for independent samples and the post hoc Tukey’s test.

When comparing G1, G2 and G3 at 14 days, there was no significant difference between G1 and G2, but there was a significant difference between G2 and G3 at 14 days. At 42 days, when comparing the formation of bone neoformation between the three groups, there was no significant difference between G1 and G2, but there was a significant difference between G1 and G3 (Figure 8 and Table 2).

## 4. Discussion

When repairing bone defects, local factors must be considered, such as the characteristics of the defect in relation to extension, location and dimensions, and systemic factors of the individual must also be considered, such as the presence of chronic diseases and harmful habits such as alcohol consumption or smoking. The use of adjuvant therapies and biomaterials, which contribute to stimulating the repair process and modulating the microenvironment [63,64], are constantly used, mainly in medical areas such as orthopedics, and dentistry, such as surgery, periodontics and implantology.

Therefore, this study evaluated the use of photobiomodulation therapy (PBM) associated with Bonefill^®^ bone xenograft (IM) and fibrin biopolymer (FB,) in bone defects surgically created in the tibia of rats. In general, the group that associated the 3 treatments (G3) obtained better rates, showing higher levels of bone maturation per period (14 and 42 days). The use of PBM favored technical repair, especially when associated with the biocomplex (bone graft + fibrin biopolymer) which, in turn, act as scaffolds that create a beneficial microenvironment for the process. The association of two three-dimensional scaffolds, such as bovine bone graft and a fibrin derivative, allows better accommodation and stability in the recipient bed, with greater vascular sprouting, essential factors for the success of a graft with bone formation [65,66,67].

Using µCT in this experiment, it was possible to observe the tissue repair process with the presence of the biomaterial up to 42 days in all groups. The analysis carried out using this method was only qualitative, as the biomaterial has a radiopacity similar to bone tissue, making it difficult to correctly measure the quantity [68,69]. However, we opted to use histological slides for this quantitative assessment, using histomorphometry, for greater precision. Qualitatively, through microtomography, it was possible to observe, according to the literature [70,71], the presence of newly formed bone tissue amidst the biomaterial particles, indicating an active repair and maturation process.

Histologically, the new bone formed was observed throughout the entire extent of the defect area (~12.56 mm^2^), around the biomaterial particles. In the FB groups (G2 and G3), it was possible to observe a greater proximity of the particles, demonstrating their benefit in incorporating the biomaterial [23]. Bone quality at 42 days is higher than at 14 days, and is expected within the bone maturation process. In G3, this maturation is more evident and the biomaterial is better integrated into the newly formed bone tissue [72,73].

At 14 days, histomorphometrically, G3 had a higher percentage of new bone formation (28.4 ± 2.3), groups G1 and G2 had lower percentages (24.1 ± 2.91; 22.2 ± 3.11), with no significant difference between them. When inserted into the defect, the biomaterials participate in an inflammatory process in which the body tries to reabsorb them and gradually remodel the local bone tissue, which is why there is a lower rate of bone formation in the first few days. The PBM associated with scaffolds makes a positive contribution by reducing the inflammatory response and stimulating the formation of new bone through its biostimulatory and local modulation effects. According to Reis et al. (2022) [74], photobiomodulation is a therapy that contributes to neovascularization, stimulating angiogenic factors, which is extremely important in the initial phase of repair.

Quantitatively, at 42 days, there was a greater presence of newly formed bone and a higher level of maturation in G3 (35.1 ± 2.55), compared to G1 and G2 (30.1 ± 2.9; 31.8 ± 3.12). During this period, the maturation process advanced, being evident in the 3 groups, but more accentuated in G3. Biomaterials help in the migration of cells for bone neoformation, operating as a framework for these cells to arrive and organize themselves. Della Colleta et al. (2022) [66], showed in their study that the FB also acts as a scaffold, facilitating the insertion and stability of the graft in place. Bueno et al. (2023) [75], demonstrated that the use of FB extends to other tissues such as nerve repairs, being a biomaterial that allows a precise and objective intervention in delicate tissues.

In a previous study, in which we used the Bonefill^®^ xenograft in bone defects in rats, which ingested water or alcohol as a liquid diet, the groups with clot filling or IM biomaterial were tested. In the clot/water group (called CCG) bone formation was 5.30 ± 3.08, 8.41 ± 5.17, 15.50 ± 7.14 and 14.51 ± 7.69, at 10, 20, 40 and 60 postoperative days, respectively. In animals of the biomaterial/water group (called BCG), the volume density of new bone was 7.54 ± 6.56, 13.79 ± 11.14, 12.97 ± 7.07 and 13.34 ± 12.45, at 10, 20, 40 and 60 days, respectively [41]. Because this study was carried out on rat calvaria, to better adapt to the site of the defect and standardize its extension [76], a trephine drill was used, instead of a spherical drill, which we use mainly in long bones such as the tibia [77].

Through the evaluation of birefringence of collagen fibers, using Picrosirius-red staining subjected to specific with polarized light, it was observed that collagen fibers are present from the beginning of the repair, are organized in parallel, thickening over time, to carry out the mineralization of the organic matrix, and thus restore, ideally, the function and local histology [78,79]. Fundamentally, the quality of the newly formed organic matrix during the bone repair process is assessed. Depending on the nature of the fibers, they are birefringent to green when more mature, yellow and red when less mature [80]. At 42 days, a more evident cell maturation was observed, with more organized collagen fibers in the G3 group (biomaterial + fibrin biopolymer + photobiomodulation), showing a more mature regeneration in relation to the other groups.

According to the study by Rosso et al. (2019) [23], in long bones of rats, photobiomodulation therapy and heterologous fibrin sealant, when used simultaneously, developed for the formation of a more mature bone, evidenced by the composition of more organized and mature collagen fibers at the end of 42 days. According to Reis et al. (2022) [70], PBM therapy acts on the process of maturation and arrangement of collagen fibers, leading to thickening and arrangement of fibers in parallel.

During bone regeneration, histologically, we observe a tissue with fibroblasts, responsible for the deposition of collagen fibers; osteoblasts, which act in the formation of the bone matrix; osteoclasts that act in bone resorption and formation of vessels that carry nutrients and cells to the site of regeneration [81,82,83]. The immunohistochemical technique detected the presence of OCN (in the connective tissue and on the surface of the newly formed bone tissue over the biomaterial particles), and TRAP (present in the osteoblasts bordering the newly formed bone tissue and in some fibroblasts). Both immunomarkers are expected in regions of revascularization and osteogenesis, in which the bone tissue undergoes the process of recomposition, until it reaches its tissue maturation [84,85,86].

At 42 days, the G1, G2 and G3 groups showed immunostaining for OCN in the newly formed bone lamellae, which would be expected in all groups, as they go through the process of bone maturation [87,88,89]. In the OCN and TRAP analysis, a higher immunostaining pattern was presented in the G1 group when compared to the others, which would be justified by the fact that the G1 group is still in a less mature bone formation process than the other groups [90,91,92].

According to a study carried out by Qin et al. (2020) [93], OCN immunostaining indicates a positive effect during the initial phases, in the production of bone matrix, and in the final phases of bone mineralization and cell renewal. Hayman [94] showed that the TRAP enzyme plays an essential role in the formation of the bone matrix, acting in the processing of type I collagen (among the main components of the bone matrix), applied as an immunomarker of osteoclastic activity, indicating the process of bone maturation.

The heterologous fibrin sealant, not derived from human blood components, did not trigger an inflammatory process, observing the absence of local inflammatory infiltrate [95]. In addition, the fibrin biopolymer, due to its potential properties, can be applied in several regenerative therapies, in a variety of tissues, such as bone, skin and nerves [96]. FB has several functions, being able to act as a scaffold, drug delivery and act in local hemostasis [97,98]. Another important point for its clinical relevance is its low manufacturing cost, which can be used in public health systems in the treatment of chronic venous lesions, being a safe and effective product for future clinical research [47].

The Bonefill^®^ xenograft (IM) showed characteristics of osteogenesis, osteoconduction and resorption, which, according to Fernandes et al. (2022) [40], are desired in bone substitutes and are found in this grafting biomaterial. IM is a Brazilian scaffold, widely known and marketed for dentistry and regenerative medicine, which has similar characteristics to the most studied and used xenograft in the world, Bio-Oss^®^ (Geistlich Pharma AG, Wolhusen, Switzerland), with similar porosity, which is identified as one of the main osteoconductive factors. In relation to the potential for new bone formation, IM presents a smaller amount of newly formed bone, but with a more advanced structural stage [99]. Taking into account the similarity between them, and the fact that the commercial value of IM is lower than that of Bio-Oss^®^, IM represents an interesting option for use [100].

In relation to laser therapy, there are several application protocols, varying in their wavelength and energy density, so there is still no consensus protocol, but several studies that used a protocol similar to the one in our study (wavelength 830 nm, optical output power 30 mW, energy density 6.2 J/cm^2^), which revealed benefits to the healing process and faster bone tissue formation [101,102,103,104,105,106]. There is still a need to expand the studies related to the use of laser photobiomodulation in order to better clarify the possible protocols and their actions.

Other physical methods also contribute to increasing bone formation, in addition to photobiomodulation, such as low-intensity pulsed ultrasound (LIPUS), in which the repair process is regulated by the electric field and bone metabolism is electrically stimulated [107,108]. There is evidence that stimuli (mechanical or electrical), of relatively low amplitude and high frequency, can influence bone formation and resorption. Therefore, they can be used clinically to inhibit or reverse bone loss, as observed in electric and electromagnetic field methods [109].

As a possible limitation of this study, we can consider the number of groups studied. The number of animals (*n*) and their random distribution were based on the principle of the 3Rs and the approval of the ethics committee, in which there is a commitment from the world scientific community to follow the principles of “reduction, substitution and refinement” in the use of animals. Therefore, it was decided not to perform groups with defects filled only with clot or autogenous bone, which have already been studied, including with the same methodology used in our experiment [110,111], focusing only on the recomposition of a defect that requires grafting, with a new combination of scaffolds (biocomplex) associated with PBM, never studied before. However, we reduced the number of animals involved, within the ethical protocol of the University in which our study was approved and carried out, focusing only on the originality of the experimental groups and study objectives [23].

To translate this study into a clinical protocol, inclusive in oral cavity, it will be necessary to carry out phase III of the fibrin biopolymer, which recently completed phase II, with its use in the treatment of chronic venous ulcers [47].

## 5. Conclusions

This study analyzed the effects of photobiomodulation therapy (PBM) on the repair process of experimentally produced bone defects in the tibia of rats, filled with mineralized inorganic bone matrix (IM) associated with fibrin biopolymer (FB). The results demonstrated that the groups with low-level laser PBM (G1 and G3) presented the highest percentages of new bone formation, contributing to the repair of defects, especially at 42 days, in the G3 group, which associated the biocomplex (IM + FB) with PBM.

## Figures and Tables

**Table 1:** Parameters used for PBM application.

Parameter	Description
Type of laser	GaAlAs (gallium-aluminum-arsenide) Manufacturer: Ibramed, Amparo, Brazil
Wavelength (nm)	830
Output power (mW)	30
Beam area (cm^2^)	0.116
Irradiance (mW/cm^2^)	258.62
Treatment time of irradiation, per point (s)	24
Number of points of the application LLLT	2 (One above and one below the defect)
Energy per point (J)	0.72
Total energy (J)	2.88
Energy density of irradiation, per point (J/cm^2^)	6.20
Application method	Positioned for laser irradiation at perpendicular incidence to the tibia
Emission mode	Continuous
Frequency	Immediately after surgery and three times a week until euthanasia

nm = nanometer; mW = milliwatts; cm = centimeter; J = Joule.

**Table 2:** Bone neoformation (%) in groups G1, G2 and G3, in experimental periods—14 and 42 days.

	14 Days	42 Days	*p*-Value
G1	24.1 ± 2.91 aAC	30.1 ± 2.9 bA	*p* = 0.0116
G2	22.2 ± 3.11 aA	31.8 ± 3.12 bAB	*p* = 0.0012
G3	28.4 ± 2.3 aBC	35.1 ± 2.55 bBC	*p* = 0.0026

Line, a ≠ b, different lowercase letters, indicate difference with statistical significance in each group in 14 and 42 days (unpaired *t*-test). Column, A ≠ B ≠ C, different capital letters, indicate difference with statistical significance in the same period between G1, G2 and G3 (ANOVA and Tukey). Values, mean ± standard deviation (*p* < 0.05).

## Data Availability

Data presented in this study are available on request from the corresponding author.
